# Supplementary material for: #WhatIEatinaDay: The Quality, Accuracy, and Engagement of Nutrition Content on TikTok
Source: Nutrients. 2025 Feb 24;17(5):781. doi: 10.3390/nu17050781 (PMC11901546; doi:10.3390/nu17050781)
Supplement: Supplementary file 1 [file nutrients-17-00781-s001.zip › nutrients-3490824-supplementary--final.pdf]

Supplementary Materials

Table S1. Full search strategy.

The following hashtags were entered into TikTok to yield nutrition-related posts for this study: #healthyfoodideas, #eatinghealthymeals, #weightlossrecipes, #healthandnutrition, #healthymealideas, #weightlossnutrition, #dietplanweightloss, #dietchallenge, #superfood, #cleaneating, #nutritiontips, #nutrition.

Table S2. Descriptions of "Nutrition Topic" criteria.

Nutrition Topic	Description
**Recipes, meal ideas and "What I Eat in a Day" (WIEIAD)**	Recipes, meal suggestions and WIEIAD for overall health, wellness or non-specified nutrition goals e.g. healthy recipes, balanced meals, whole foods, clean eating etc.
**General nutrition advice**	Nutrition information and advice for overall health, wellness or non-specified nutrition goals e.g. food group information, healthy fats, why a specific food should be avoided etc.
**Food, nutrient and supplement information**	Nutrition advice or information extolling the health benefits of a specific food, nutrient or supplement without a specific focus on health goals e.g. green tea, superfoods, green powders etc.
**Weight loss**	Recipes, meal suggestions, WIEIAD, nutrition information and advice predominately linked to weight loss e.g. Weight loss recipes, low calorie recipes, weight loss and fasting strategies, weight loss stories etc.
**Increased protein intake/muscle gain**	Recipes, meal suggestions, WIEIAD, nutrition information and advice predominately linked to high protein intake, promoting muscle gain or fitness goals e.g. high protein recipes, increased protein strategies, muscle gain strategies etc.
**Specialised nutrition**	Recipes, meal suggestions, WIEIAD, nutrition information and advice towards specific demographics with unique nutritional requirements e.g. paediatrics, oncology, geriatric, sports performance and chronic illnesses.
**Goal-orientated nutrition advice**	Recipes, meal suggestions, WIEIAD, nutrition information and advice predominately linked to a specific health goal (excluding weight loss, increased protein intake and muscle gain). Goals can include gut health, skin and hair health, mental health, sleep quality, hormone balance, anti-inflammation and sexual health etc.
**Dietary patterns**	Recipes, meal suggestions, WIEIAD, nutrition information and advice predominately linked to specific dietary pattern but with non-specified nutrition goals. Example dietary patterns can include vegetarian/vegan, keto, paleo, carnivore, WOMAD, Whole30, 75 Hard etc.
**Other**	Nutrition-related content that does not fit into the above categories e.g. parodying diet culture.

Table S3. Descriptions of "Content Creator Type" criteria.

Content Creator Type	Description
**Health or wellness content creators**	Content creators with no medical or health credentials with content primarily consisting of providing health and dietary advice. Includes weight loss coaches, diet coaches, holistic nutritionist, personal accounts recounting weight loss journeys, wellness shop owners, supplement brand owners etc.
**Fitness content creator**	Content creators whose content primarily consist of fitness or workout content. Can include fitness coaches, fitness trainers etc.
**Dietitian**	Accredited Practising Dietitians (AU), Registered Dietitians (RD).
**Nutritionist**	At minimum a Bachelor of Nutrition or current Dietitian students.
**Other health professionals**	Other accredited health or medical professionals including doctors, physicians, medical specialists, nurses, psychologists, physiologists etc.
**Food content creator**	Content creators mainly posting recipes. Can include those specialising in certain diets (high protein, low calorie, gluten-free, vegan, vegetarian, keto, paleo etc.) and chronic conditions (IBS, diabetes) so long as content is primarily recipe-based without health/nutrition advice.
**Content farm**	Accounts that produce large amounts of low-quality health and nutrition information to generate traffic and ad revenue. Telltale signs include extensive reposting of content, extensive usage of hashtags, and usage of AI tools for text or voiceovers.
**Other**	Accounts whose content is mostly unrelated to health, nutrition or food. Includes: lifestyle content creators, personal accounts etc.

Table S4. Accuracy Assessment Categories.

Accuracy Categories	Description
**Completely Accurate**	Fully aligned with evidence-based dietary guidelines.
**Mostly Accurate, Some Inaccuracies**	Largely aligned with scientific evidence but containing minor misleading claims or lacking context.
**Mostly Inaccurate, Some Accurate Information**	Predominantly inaccurate but with some correct elements.
**Completely Inaccurate**	Contradictory to evidence-based guidelines and potentially harmful.
**Not Assessable**	Lacking sufficient detail to determine accuracy.

Table S5. Level of Evidence Classification.

Level of Evidence	Description
**Level A (Strong Evidence)**	Based on systematic reviews or national dietary guidelines.
**Level B (Moderate Evidence)**	Backed by multiple peer-reviewed studies but not yet reflected in dietary guidelines.
**Level C (Limited Evidence)**	Based on single studies or emerging research.
**Level D (Weak Evidence)**	Lacking scientific backing, anecdotal claims, or unverified information.

Table S6. ANOVA report for engagement metrics of nutrition-related TikTok posts categorised by levels of accuracy.

Engagement Metric	Source of Variation	SS	df	MS	F	P-value	F-crit
**Comments**	Between Groups	1.82E+11	4	4.55E+10	0.831	0.507	2.408
	Residuals	1.34E+13	245	5.48E+10			
	Total	1.36E+13	249				
**Likes**	Between Groups	7.71E+10	4	1.93E+10	1.021	0.314	2.408
	Residuals	8.68E+12	245	3.54E+10			
	Total	8.76E+12	249				
**Shares**	Between Groups	1.31E+09	4	3.27E+08	0.49	0.743	2.408
	Residuals	1.63E+11	245	6.67E+08			
	Total	1.65E+11	249				
**Saves**	Between Groups	1.08E+10	4	2.70E+09	0.341391	0.849855	2.408
	Residuals	1.93E+12	245	7.90E+09			
	Total	7.90E+09	249				

Table S7. ANOVA report for engagement metrics of nutrition-related TikTok posts categorised by levels of evidence.

Engagement Metric	Source of Variation	SS	df	MS	F	P-value	F-crit
**Comments**	Between Groups	2.31E+06	4	5.79E+05	0.368	0.831	2.408
	Residuals	3.85E+08	245	1.57E+06			
	Total	3.87E+08	249				
**Likes**	Between Groups	1.68E+11	4	4.21E+10	0.767	0.548	2.408
	Residuals	1.34E+13	245	5.49E+10			
	Total	1.36E+13	249				
**Shares**	Between Groups	2.55E+09	4	6.38E+08	0.768	0.547	2.408
	Residuals	2.04E+11	245	8.31E+08			
	Total	2.06E+11	249				
**Saves**	Between Groups	3.52E+10	4	8.81E+09	1.130	0.343	2.408
	Residuals	1.91E+12	245	7.80E+09			
	Total	1.95E+12	249				
